# Aged Nicotinamide Riboside Kinase 2 Deficient Mice Present an Altered Response to Endurance Exercise Training

**DOI:** 10.3389/fphys.2018.01290

**Published:** 2018-09-19

**Authors:** Robin Deloux, Cynthia Tannous, Arnaud Ferry, Zhenlin Li, Mathias Mericskay

**Affiliations:** ^1^Signalling and Cardiovascular Pathophysiology—UMR-S 1180, Univ. Paris-Sud, INSERM, Université Paris-Saclay, Châtenay-Malabry, France; ^2^Department of Biology of Adaptation and Ageing, CNRS UMR8256, INSERM U1164, Institute of Biology Paris-Seine, DHU FAST, Sorbonne Universités, Paris, France; ^3^Sorbonne Paris Cité, Université Paris Descartes, Paris, France; ^4^Institut de Myologie, UMR-S 794, INSERM, CNRS, Sorbonne Universités, Paris, France

**Keywords:** heart, skeletal muscle, aging, NAD, NMRK2, exercise, endurance training

## Abstract

**Background:** Skeletal muscle aging is marked by the development of a sarcopenic phenotype, a global decline of muscle energetic capacities, and an intolerance to exercise. Among the metabolic disorders involved in this syndrome, NAD metabolism was shown to be altered in skeletalmuscle, with an important role for the NAMPT enzyme recycling the nicotinamide precursor. An alternative pathway for NAD biosynthesis has been described for the nicotinamide riboside vitamin B3 precursor used by the NMRK kinases, including the striated muscle-specific NMRK2.

**Aim:** With this study, our goal is to explore the ability of 16-month-old *Nmrk2*^−/−^ mice to perform endurance exercise and study the consequences on muscle adaptation to exercise.

**Methods:** 10 control and 6 *Nmrk2*^−/−^ mice were used and randomly assigned to sedentary and treadmill endurance training groups. After 9 weeks of training, heart and skeletal muscle samples were harvested and used for gene expression analysis, NAD levels measurements and immunohistochemistry staining.

**Results:** Endurance training triggered a reduction in the expression of Cpt1b and AcadL genes involved in fatty acid catabolism in the heart of *Nmrk2*^−/−^ mice, not in control mice. NAD levels were not altered in heart or skeletal muscle, nor at baseline neither after exercise training in any group. *Myh7* gene encoding for the slow MHC-I was more strongly induced by exercise in *Nmrk2*^−/−^ mice than in controls. Moreover, *IL*-15 expression levels is higher in *Nmrk2*^−/−^ mice skeletal muscle at baseline compared to controls. No fiber type switch was observed in plantaris after exercise, but fast fibers diameter was reduced in aged control mice, not in *Nmrk2*^−/−^ mice. No fiber type switch or diameter modification was observed in soleus muscle.

**Conclusion:** In this study, we demonstrated for the first time a phenotype in old *Nmrk2*^−/−^ mice in response to endurance exercise training. Although NMRK2 seems to be predominantly dispensable to maintain global NAD levels in heart and skeletal muscle, we demonstrated a maladaptive metabolic response to exercise in cardiac and skeletal muscle, showing that NMRK2 has a specific and restricted role in NAD signaling compared to the NAMPT pathway.

## Introduction

Skeletal muscles are highly plastic organs that adapt their shape and performance to the physiological demand. In a simplified way, resistance training leads to hypertrophy of fibers while endurance training stimulates oxidative metabolism in fibers. This plasticity however is progressively lost with aging, which is associated both with a reduction in muscle mass and strength at baseline (sarcopenia) and a reduced gain in performance and muscle remodeling in response to resistance exercise (Kumar et al., 2009). Muscle aging is also associated with reduced endurance exercise capacity that is attributed not only to decreased cardiovascular performance but also to an intrinsic decrease in the quality and quantity of muscle cell mitochondria (Lanza and Nair, 2010; Johnson et al., 2013). Hence, sarcopenic muscles are supposed to differ in the various biochemical pathways involved in exercise response (Ziaaldini et al., 2017). Yet, despite these limitations, regular exercise remains one of the most effective strategy to counteract sarcopenia and lower chronic inflammatory cytokines overload although optimization of the protocols and a better understanding of the mechanisms involved in the lower response of sarcopenic muscles resistance training are still required (Cruz-Jentoft et al., 2014; Denison et al., 2015; Ziaaldini et al., 2017; Monteiro-Junior et al., 2018).

Among the molecular mechanisms involved in the aging process, alterations in the metabolism of the Nicotinamide Adenine Dinucleotide (NAD^+^) has been shown to play a major role in most tissues, including heart and skeletal muscles (Mericskay, 2016; Yoshino et al., 2018).

NAD has the particularity of being both a coenzyme and a signaling molecule. As a coenzyme, NAD is recycled from oxidized NAD^+^ to reduced NADH in the oxido-reduction reactions of the energy metabolism, without net consumption of the total NAD pool in these processes. As a signaling molecule, NAD is a co-substrate molecule hydrolyzed by several cellular enzymes, notably the sirtuins (SIRT) deacetylases, the polyADPribose polymerases (PARP) and the CD38 and CD157 ADPribose cyclases (Mericskay, 2016; Yoshino et al., 2018). These pathways act as sensors of energetic and redox state to regulate energy metabolism (SIRT1, SIRT3), oxidative stress response and cell survival (PARP1) and Ca^2+^ signaling (CD38) in all cell types. In aged mouse skeletal muscle, there is more than 50% decline in NAD^+^ levels that alters SIRT1 activity and reduced the level of nuclear and mitochondrial encoded mitochondrial proteins (Gomes et al., 2013).

The sirtuins, PARP1 and CD38/CD157 proteins all cleave the NAD^+^ into nicotinamide (NAM) and adenine diphosphate-ribose (ADPR) for their enzymatic activities. Different salvage pathway exist that compensate for this cellular consumption of NAD^+^ (Mericskay, 2016; Yoshino et al., 2018). A major pathway in muscle is initiated by the nicotinamide phosphoribosyl transferase (NAMPT). NAMPT uses the NAM derived from the activity of NAD^+^ consuming enzymes but also nutritional NAM (vitamin B3), to generate nicotinamide mononucleotide (NMN) that is then fused to the ADP moiety of ATP by the nicotinamide adenilyl transferases (NMNAT, 1 to 3) to form the dinucleotide. Genetic depletion of the NAMPT enzyme in adult skeletal muscles leads to fiber degeneration and progressive loss of both muscle strength and treadmill endurance (Frederick et al., 2016). The contribution of the deamidated precursors, nicotinic acid and tryptophan to NAD^+^ synthesis is minimal in skeletal muscles (Mori et al., 2014).

More recently, an alternative pathway for the synthesis of NMN was discovered based on the phosphorylation of the nucleoside form of the NAM base, the nicotinamide riboside (NR), by the NR kinases NRK1 and NRK2 (official symbol NMRK1 and NMRK2; Bieganowski and Brenner, 2004; Belenky et al., 2007). NMRK2 is expressed predominantly in skeletal muscles whereas NMRK1 seems to be ubiquitously expressed but more abundant in liver and kidneys (Ratajczak et al., 2016; Fletcher et al., 2017). *Nmrk2*^−/−^ mice (12–14 weeks) showed no alteration in total NAD levels in skeletal muscles and no phenotype at baseline or after 6 weeks of endurance exercise protocol as regard muscle fiber cross-sectional area and muscle fiber type distribution (Fletcher et al., 2017).

Considering that NAD metabolism becomes crucial during aging, we explored in the present study the ability of middle-age *Nmrk2*^−/−^ mice (16 month) to perform endurance exercise training and the impact of this training on muscle fiber cross-sectional area, muscle fiber type distribution and myokines expression in different muscles.

## Materials and methods

### Animals and endurance training

All experiments with animals conformed to the Directive 2010/63/EU of the European Parliament and were approved by the ethics committee Charles Darvin #5 and authorized by the Ministry of Research in application of the French Rural Code, notably the articles R. 214-87 to R. 214-126 (agreement 00369.03: Role of the kinase NMRK2 in skeletal muscles). The animal experiments were performed in the UPMC university Center for Experimental Exploration, UMS 028, 105 Boulevard de l'Hôpital, Paris 75013, holding the agreement # A751315.

Constitutive *Nmrk2* knocked-out mice were obtained and genotyped in a C57BL/6NTac genetic background as previously described (Vaur et al., 2017). Homozygous *Nmrk2*^−/−^ mice were viable and fertile. For this study, 16 sixteen-month old female mice were used (*n* = 10 C57BL/6N wild-type mice and *n* = 6 *Nmrk2*^−/−^ mice). Half of the animals were then randomly assigned to sedentary or endurance training groups (*n* = 5 C57BL/6N wild-type mice and *n* = 3 *Nrmk2*^−/−^ mice for each condition). Mice were acclimated to the treadmill for 3 consecutive days, and first submitted to an exercise performance test involving a warm up phase at 5 to 15 cm/s for 15 min, followed by an acute exercise phase where the speed of the treadmill was progressively increased until reaching signs of exhaustion (more than 5 shocks within 60 s, or more than 5 consecutive seconds on the shock grid with attempting to reengage on the treadmill). Endurance training protocol was carried out for 9 weeks and was composed of a warm up phase at 5 to 15 cm/s for 15 min followed by 0 to 45 min at 15 cm/s and after that 45 min in which speed was progressively increased from 15 to 30 cm/s. Protocol was strictly identical for every animal used in this study.

### RT-qPCR analysis

Total RNA were extracted from tissue samples using TRI Reagent® (Molecular Research Center) and a tissue homogenizer (Precellys®, Bertin Instruments) following the manufacturer instructions. RNA concentrations were quantified by spectrophotometry using NanoDrop 2000 (Thermo Fisher Scientific). cDNAs were reversed transcripted from 2 μg of RNA using the iScript™ Reverse Transcription kit (Biorad). Quantitative PCR reactions were carried out on CFX™ Real-Time PCR Detection System (Biorad), in triplicates for each sample in a 7.5 μL volume containing 3.75 μL of SsoAdvanced™ Universal SYBR® Green Supermix, 0.5 μM of each forward and reverse primers and 2.5 μL of 1:20 diluted cDNA. Primers sequences used in this study are available on request. The expression of *Hprt* (Hypoxanthine-guanine phosphoribosyltransferase) gene or the mean of the expression of *Rplp0* (Ribosomal Protein, Large, P0) and *Ywhaz* (Tyrosine 3-Monooxygenase/Tryptophan 5-Monooxygenase Activation Protein Zeta) genes were used as a reference for normalization in heart and gastrocnemius muscle, and plantaris and soleus muscles, respectively.

### NAD extraction and quantification

Metabolites were extracted by homogenizing tissue samples in a buffered ethanol solution (75% ethanol/25% HEPES 10 mM pH 7.1, 10 μL/mg of tissue) with a tissue homogenizer (Precellys®, Bertin Instruments). Extracts were heated at 80°C for 5 min, chilled on ice and centrifuged for at 15000 g for 15 min at 4°C. NAD levels were quantified using an MTT-formazan recycling assay. Samples extracts were diluted in water to a final volume of 25 μL. After adding 100 μL of reaction buffer (600 mM ethanol, 0.5 mM 3-(4.5-dimethylthiazol-2-yl)-2.5-diphenyltetrazolium bromide (MTT), 2 mM phenazine ethosulfate (PES), 120 mM Bicine (pH7.8), yeast alcohol dehydrogenase 0.05 mg/mL (SIGMA A3263), kinetics of the reaction was assessed by measuring OD at 550 nm every 30 s for 40 min using a TECAN Infinite F500 microplate reader. Samples NAD concentrations were determined by comparing the slope of the reaction (OD/s) to a range of standard NAD^+^ concentrations.

### Immunofluorescent staining

Immediately after sacrifice, fast (plantaris) and slow-twitch (soleus) muscles where embed in Tissue-Tek (Sakura, USA) and frozen in liquid nitrogen-cooled isopentane at −150°C. They when then stored at −80°C and sliced into 8 μm cryosections with CM1860 Cryostat, (Leica). Immunohistochemical staining of Myosin Heavy Chain (MHC) isoforms was performed using mouse monoclonal antibodies BAD5 (MHC-I specific, alternate name β-MHC, IgG2b, 1:100 dilution), SC-71 (MHC-IIA specific, IgG1, 1:100 dilution) and BF-F3 (MHC-IIB specific, IgM, 1:100 dilution) respectively, obtained from Developmental Studies Hybridoma Bank (DSHB, University of Iowa). Laminin staining was also performed for labeling basement membranes using a rabbit polyclonal antibody (L9393, Sigma). Briefly, muscle sections were fixed with 4% PFA for 5 min, washed twice (5 min each) in PBS, permeabilized in 0.1% Triton for 10 min, washed twice in PBS, saturated in 5% IgG-free BSA for 45 min, washed once in PBS, incubated with 1:100 mouse FAB for 20 min, washed once in PBS. Primary antibodies were then incubated overnight at 4°C. After washing 3 times in 0.1% Tween20 in PBS, secondary antibodies were used to selectively bind to each primary antibody: goat anti-mouse IgG2b conjugated with Alexa Fluor® 350, goat anti-mouse IgG1 conjugated with Alexa Fluor® 555, goat anti-mouse IgM conjugated with Alexa Fluor® 488, and goat anti-rabbit conjugated with Alexa Fluor® 633, all diluted at 1:400 in 5% IgG-free BSA. After 3 washes in 0.1% Tween20 in PBS, muscle section were mounted using 70% glycerol. Pictures were taken using and inverted fluorescence microscope (DMi8, Leica). Pure MHC-IIX fibers were not stained by these antibodies and appeared black. Fiber type distribution and MinFeret were assessed using ImageJ software.

### Statistical analysis

Animals were assigned to sedentary or endurance training groups by randomization. Shapiro–Wilk test were applied to test for normality of distribution before to use parametric test. To assess statistical significance between control and *Nmrk2*^−/−^ mice at exhaustion test, *t*-tests for independent samples were performed. For RT-qPCR analysis, NAD levels and fiber type and MinFeret comparisons, two-way ANOVA for independent samples were performed, followed by *post-hoc* Tukey tests for multiple comparison when an interaction between the genotype and the endurance training factors was established. Global MinFeret distribution statistical differences were assessed using Chi-square test. Values are expressed as mean ± SEM.

## Results

### Endurance training and muscle performance in *Nmrk2*^−/−^ mice

**Figure 1:**
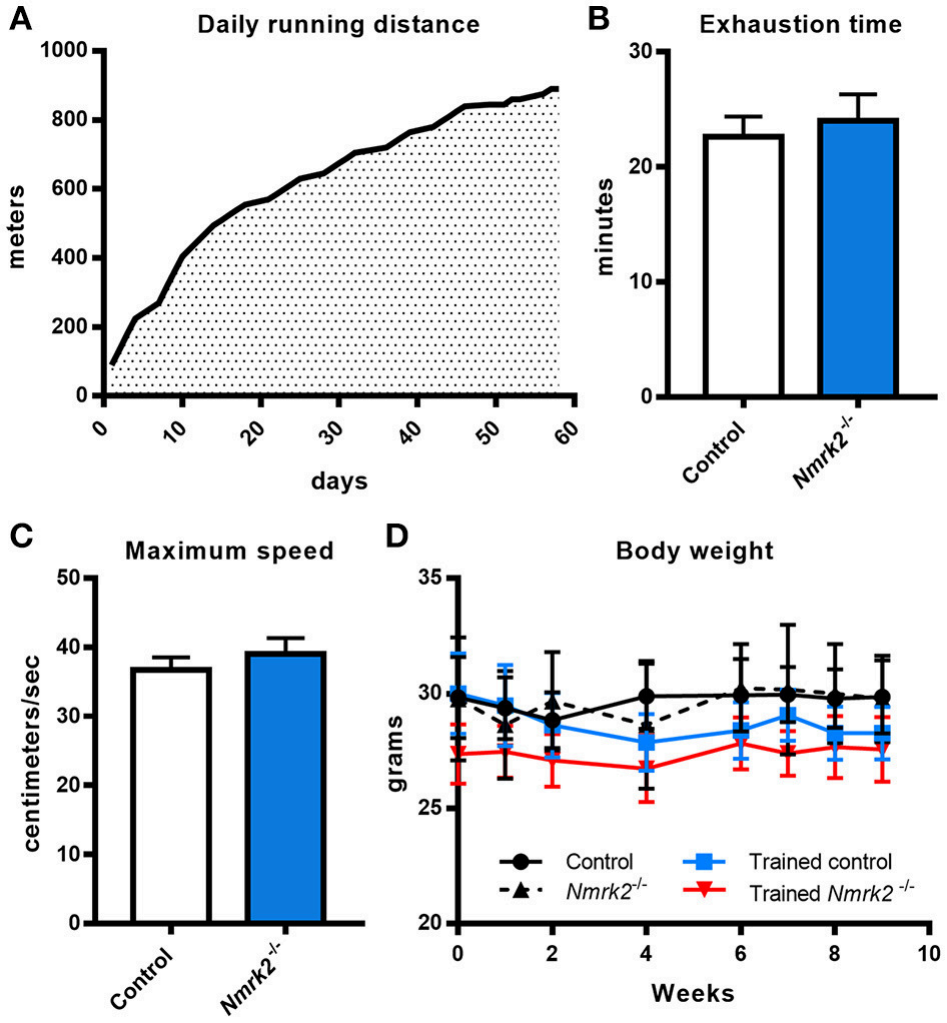
Daily running distance during endurance training, endurance test and body weight evolution. **(A)** Evolution of the daily running distance imposed to control and *Nmrk2*^−/−^ mice during the 9 weeks of endurance training. **(B, C)** Maximum speed **(B)** and time **(C)** reached at exhaustion during endurance test**. (D)** Evolution of body weight along the training protocol. *N* = 5 in each sedentary and trained control group, and *N* = 3 in each sedentary and trained Nmrk2^−/−^ group. Statistical analysis: **(B,C)**, *t*-test for independant samples. **(D)** two-way ANOVA for independent samples.

Considering the old age of the mice, 16 months at the beginning of the endurance training protocol, and the potential effect of *Nmrk2* deficiency on exercise capacities, training protocol was adapted by starting at a very low speed (15cm/s, 100 m run per session) that was progressively increased to reach 30 cm/s (890 m per session) at the end of the protocol. All animals were able to follow the complete training (Figure 1A). To assess if there was any difference in term of exercise capacity and endurance between control and *Nmrk2*^−/−^ mice, animals were submitted to a treadmill exhaustion test 1 week after the beginning of the protocol. *Nmrk2* ^−/−^ mice reached exhaustion after 24.0 ± 2.3min vs. 22.6 ± 1.8 min for control mice (Figure 1B), at a speed of 39.0 ± 2.3 cm/s *vs*. 36.7 ± 1.8 cm/s for control mice (Figure 1C), with no statistical difference between the groups. After 9 weeks of training, there was no significant body weight change in trained control and *Nmrk2*^−/−^ mice (Figure 1D).

### Effects of endurance training on cardiac gene expression and NAD levels

**Figure 2:**
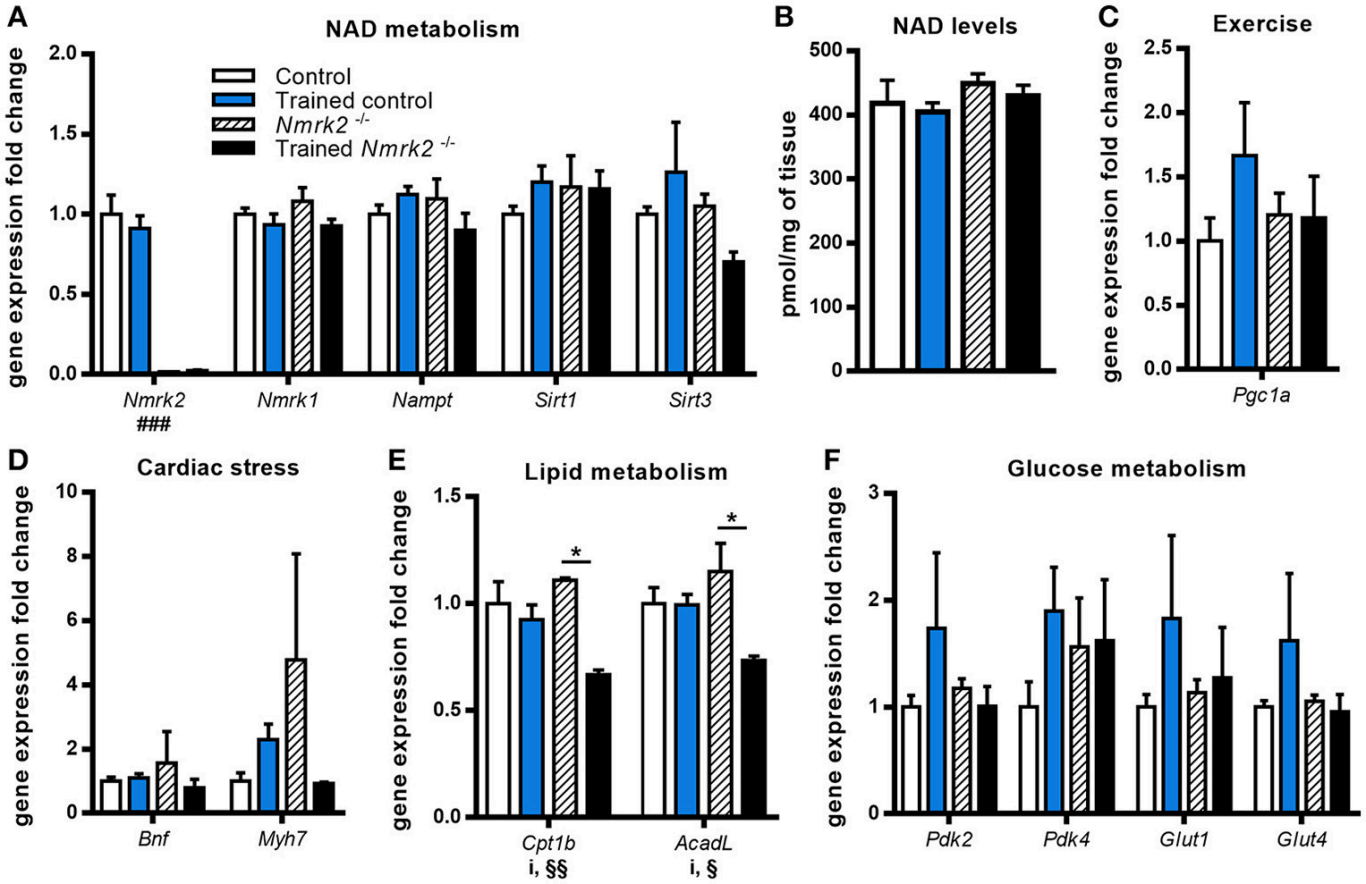
Gene expression analysis and global NAD levels in left ventricle. **(A)** Relative quantification by RT-qPCR of *Nmrk1, Nmrk2, Nampt, Sirt1*, and *Sirt3* mRNA levels in left ventricule. **(B)** Myocardial NAD levels. **(C–F)** Relative quantification by RT-qPCR of *Pgc1*α **(C)**, *Bnf* and *Myh7* **(D)**, *Cpt1b* and *AcadL* **(E)**, *Pdk2, Pdk4, Glut1*, and *Glut4* **(F)** mRNA levels in left ventricule. *n* = 5 in control and trained control groups, *n* = 3 in *Nmrk2*^−/−^ and trained *Nmrk2*^−/−^ groups. Results are expressed as mean values ± SEM. Statistical analysis: two-way ANOVA for independent samples. ^i^*p* < 0.05 for interaction, ^###^*p* < 0.001 for genotype effect, ^§^*p* < 0.05 and ^§§^*p* < 0.01 for endurance training effect. *p<0.05 from Tukey's multiple comparisons test when applicable.

To gain insight on NAD metabolism modulation in heart by exercise in old mice, we first analyzed the gene expression profile of several main enzymes responsible for its biosynthesis and consumption. *Nmrk2* gene expression was absent as expected in *Nmrk2*^−/−^ mice, but not modified by training in control mice. *Nmrk1* and *Nampt* expressions were not modulated by endurance training in control and in *Nmrk2*^−/−^ mice, neither was the expression of the sirtuins *Sirt1* and *Sirt3* (Figure 2A). In line with those observations, we did not observe a difference regarding NAD levels in cardiac muscle (Figure 2B). *Pgc1a*, known to be activated in the heart by exercise, was slightly increased without reaching statistical significance, when there was no induction in *Nmrk2*^−/−^ mice (Figure 2C). Cardiac stress marker *Bnf* was not clearly modulated by exercise or lack of NMRK2 (Figure 2D). The *Myh7* gene encoding the slow β-MHC was increased in only one sedentary *Nmrk2*^−/−^ mouse that did not show any other major diffference in other tested genes in the heart and skeletal muscle compared to the rest of the group. Interestingly, *Cpt1b* and *AcadL* genes, involved in fatty acids catabolism, were significantly lowered by exercise in *Nmrk2*^−/−^ mice, but not control mice (*p* < 0.05, Figure 2E). No clear effects could be identified on *Pdk2* and *Pdk4* expression, two isoforms of PDK, which negatively regulates glycolysis, neither on glucose transporters *Glut1* and *Glut4* (Figure 2F).

### Effects of endurance training on skeletal muscle gene expression and NAD levels

**Figure 3:**
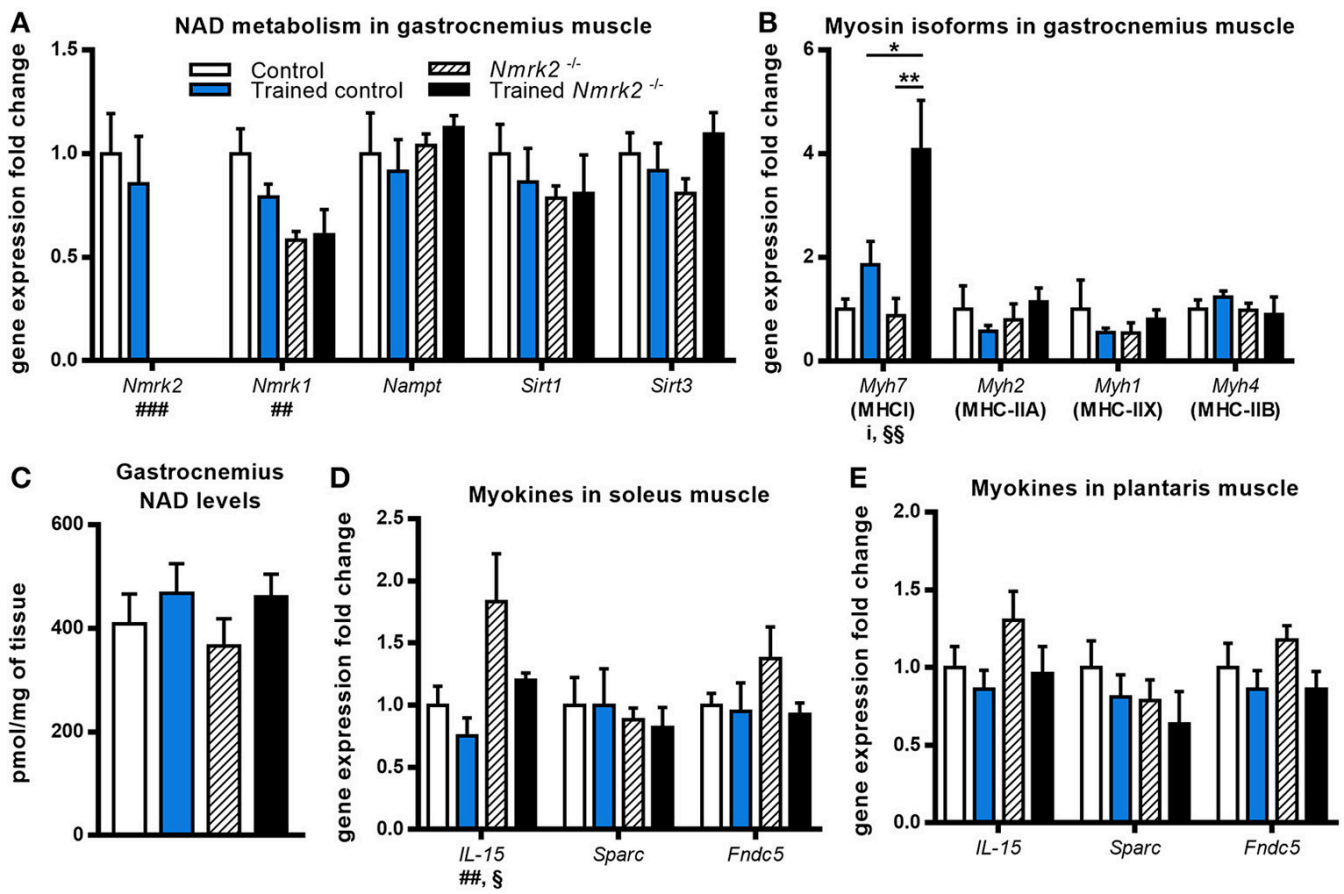
Gene expression analysis and global NAD levels in skeletal muscle**. (A,B)** Relative quantification by RT-qPCR of *Nmrk1, Nmrk2, Nampt, Sirt1, Sirt3* **(A)**, and *Myh7, Myh2, Myh1*, and *Myh4* myosin isoforms **(B)** mRNA levels in gastrocnemius muscle. **(C)** Gastrocnemius NAD levels. **(D,E)** Relative quantification by RT-qPCR of *IL-15, Sparc*, and *Fndc5* myokines mRNA levels in soleus **(D)** and plantaris muscle **(E)** respectively. Results are expressed as mean values ± SEM. Statistical analysis: two-way ANOVA for independent samples. ^i^*p* < 0.05 for interaction, ^##^*p* < 0.01 and ^###^*p* < 0.001 for genotype effect, ^§^*p* < 0.05 and ^§§^*p* < 0.01 for endurance training effect. *p < 0.05 and ^**^p < 0.01 from Tukey's multiple comparisons test when applicable.

*Nmrk2* being highly expressed in skeletal muscle, we also investigated NAD metabolism modulation enzymes in gastrocnemius muscle. No effect was observed regarding *Nampt* or *Sirt1* and *Sirt3* genes expression, but *Nmrk1* was reduced in *Nmrk2*^−/−^ mice in comparison to controls (genotype effect, *p* < 0.01, Figure 3A). In parallel with these observations, no major modulation of total NAD levels was observed (Figure 3C). We also studied the expression levels of myosin heavy-chain isoforms, known to be modulated by endurance training in young mice. Interestingly, *Myh7* gene coding for the slow MHC-I isoform was strongly induced by exercise in *Nrmk2*^−/−^ mice (*p* < 0.01, Figure 3B), to a higher level compared to trained control mice (*p* < 0.05) when no statistically significant effect of exercise was found in control mice. No modulation of other MHC isoforms gene expression was observed (*Myh2* for MHC-II1, *Myh1* for MHC-IIX or *Myh4* for MHC-IIB).

Considering the emerging role of skeletal muscle in autocrine and paracrine signaling, we also analyzed the expression of several myokines in slow (soleus) and fast-twitch (plantaris) muscles. In soleus muscle, an effect of training and genotype was observed for *IL-15* expression, a myokine that mediates muscle-fat crosstalk, seems to be higher in trained *Nmrk2*^−/−^ mice but no interaction could be identified (Figure 3D). *Sparc*, a myokine linked to muscle atrophy, and *Fndc5*, a precursor of irisin correlated to browning of adipose tissue, were not modified by exercise, even in control animals. No modifications of the expression of these 3 genes was observed in plantaris muscle although the global profile was strikingly similar to the one observed in the soleus muscle (Figure 3E).

### Modulation of MHC isoforms distribution and minferet diameter in fast and mixed muscles

**Figure 4:**
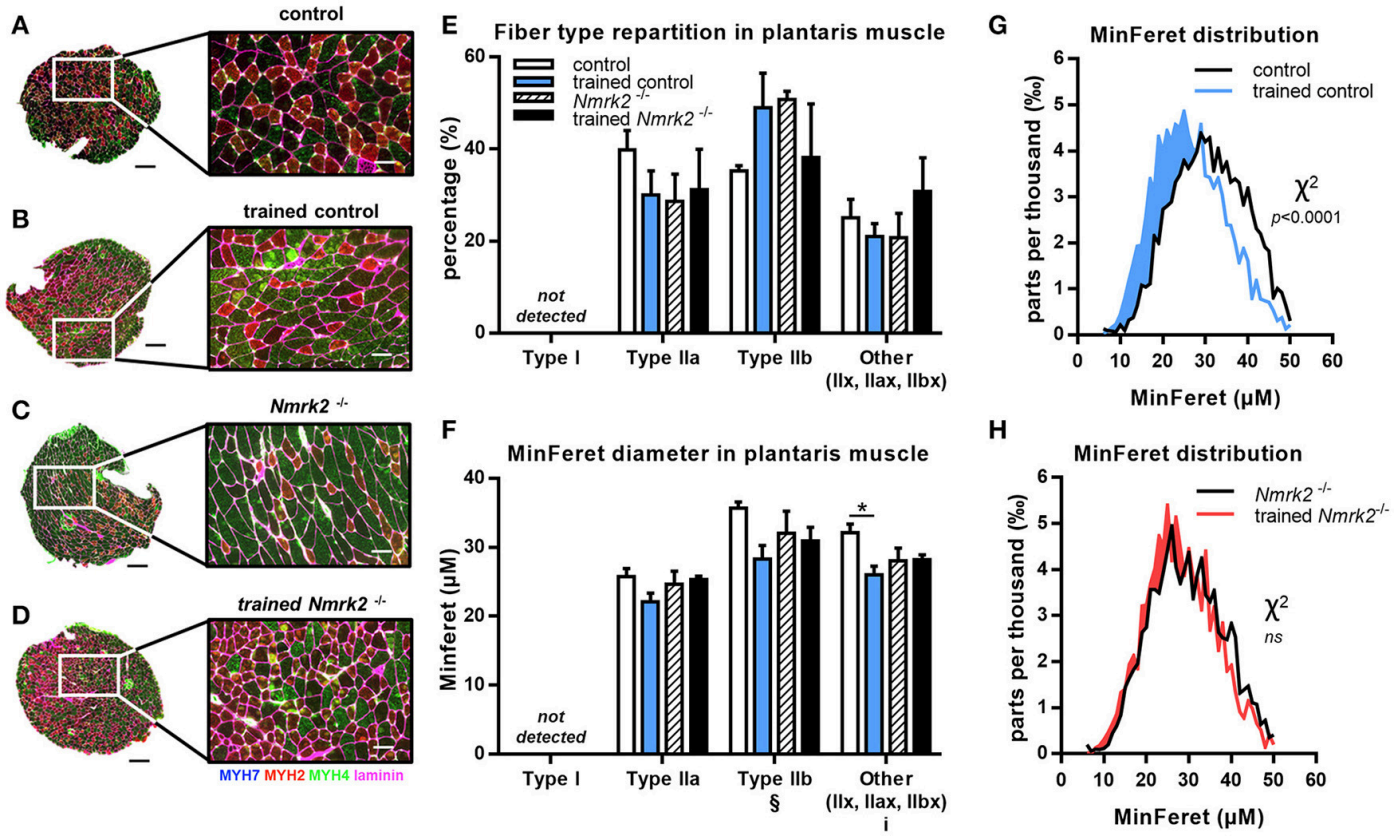
MHC isoforms distribution and MinFeret diameter in plantaris muscle. **(A-D)** Myosin isoforms and laminin immunofluorescence histochemistry (MHC-I (MYH7) in blue, MHC-IIA (MYH2) in red, MHC-IIB (MYH4) in green, laminin in magenta) on 8-μm transversal cryosections of plantaris muscle from 18 weeks old mice, WT or *Nmrk2*^−/−^, sedentary or trained. Scale bars = 200 μm for whole muscle images, 60 μM for magnified images. **(E)** Fiber type proportions calculated from whole muscle sections. Fibers negative or mixed for Type I, IIA, or IIB MHC isoforms where assigned to a separated category, in comparaison to pure MHC isoforms positive fibers. **(F)** Fibers MinFeret diameter (μM). **(G,H)** Global MinFeret distributions whithin control and trained control mice **(G)**, and *Nmrk2*^−/−^ and trained *Nmrk2*^−/−^ mice **(H)**. *n* = 5 in control and trained control groups, *n* = 3 in *Nmrk2*^−/−^ and trained *Nmrk2*^−/−^ groups. Results are expressed as mean values ± SEM. Statistical analysis **(E, F)**, two-way ANOVA for independent samples. ^i^*p* < 0.05 for interaction, ^§^*p* < 0.05 for endurance training effect. ^*^p < 0.05 from Tukey's multiple comparisons test when applicable. **(G,H)**, Chi-square test for MinFeret distribution comparisons.

To go further in the understanding of the effects of NMRK2 deficiency on skeletal muscle adaptation to exercise, we analyzed the distribution of MHC isoforms and minimal Feret diameter of fibers in fast plantaris muscle and the mixed more oxidative soleus muscles by immuno-histochemical staining. In plantaris muscle, no fiber type switch with exercise could be detected (Figures 4A–E). As expected, no type I fibers were detected in this fast muscle. MinFeret diameter trend to be reduced with exercise in control mice, reaching statistical significance for MHC-IIX fibers, when no changes were observed in *Nmrk2*^−/−^ mice (Figure 4F). Analysis of MinFeret diameter distributions of all muscle fibers showed a statistically significant shift of distribution toward thinner muscle fibers with training in control mice (*p* < 0.0001, Figure 4G), when no effect of training was observed with *Nmrk2*^−/−^ mice (Figure 4H).

**Figure 5:**
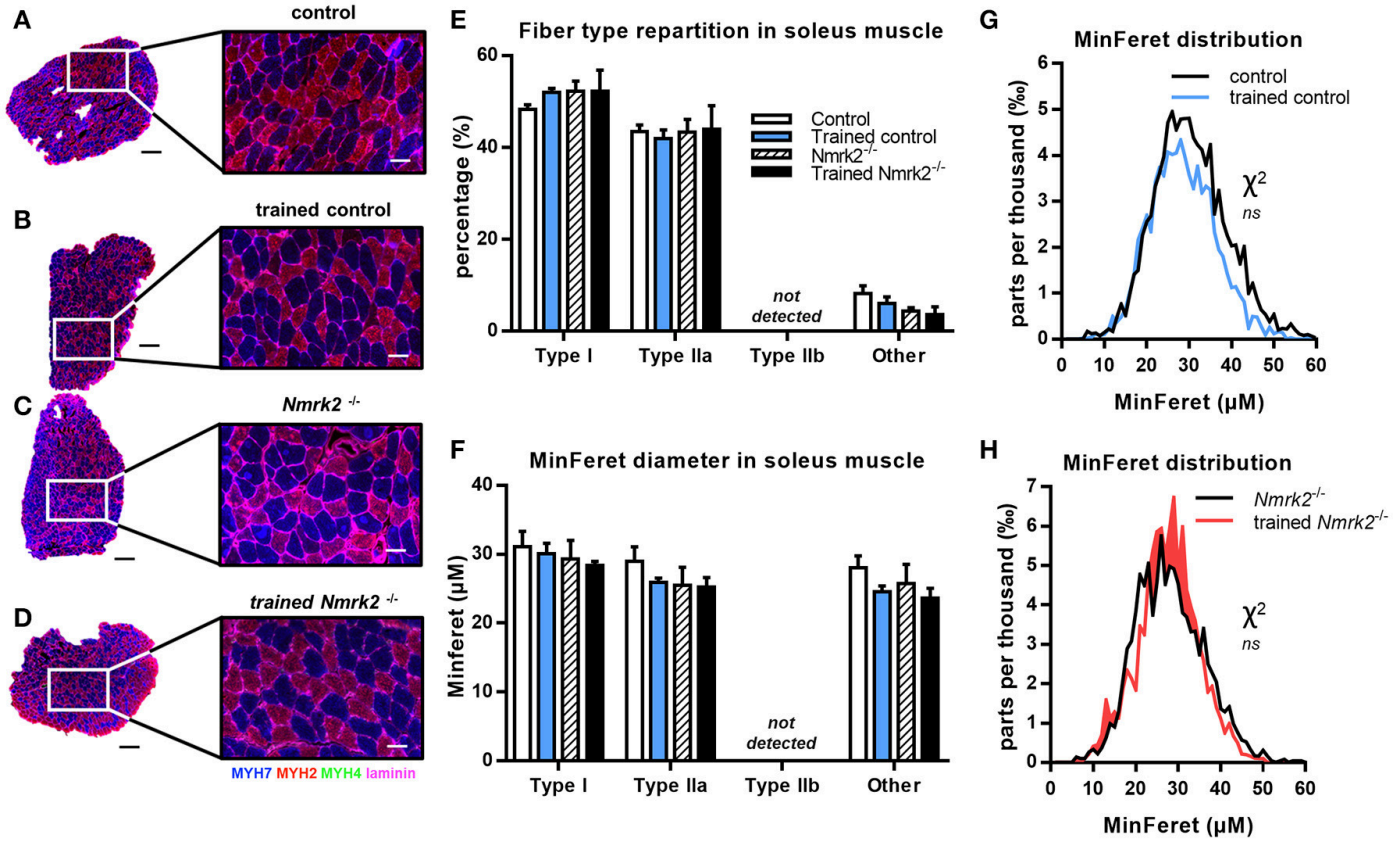
MHC isoforms distribution and MinFeret diameter in soleus muscle**. (A–D)** Myosin isoforms and laminin immunofluorescence histochemistry [MHC-I (MYH7) in blue, MHC-IIA (MYH2) in red, MHC-IIB (MYH4) in green, laminin in magenta] on 8-μm transversal cryosections of soleus muscle from 18 weeks old mice, WT or *Nmrk2*^−/−^, sedentary or trained. Scale bars = 200 μm for whole muscle images, 60 μM for magnified images. **(E)** Fiber type proportions calculated from whole muscle sections. Fibers negative or mixed for Type I, IIA, or IIB MHC isoforms where assigned to a separated category, in comparaison to pure MHC isoforms positive fibers. **(F)** Fibers MinFeret diameter (μM). **(G,H)** Global MinFeret distributions whithin control and trained control mice **(G)**, and *Nmrk2*^−/−^ and trained *Nmrk2*^−/−^ mice **(H)**. *n* = 5 in control and *n* = 4 in trained control groups, *n* = 3 in *Nmrk2* ^−/−^ and trained *Nmrk2*^−/−^ groups. Results are expressed as mean values ± SEM. Statistical analysis **(E,F)**, two-way ANOVA for independent samples. **(G,H)**, Chi-square test for MinFeret distribution comparisons.

In the soleus muscle, there was no difference in term of fiber type repartition between control and *Nmrk2*^−/−^ mice, and no fiber type switching was observed with exercise in any group (Figures 5A–E). Note that no Type IIb fast twitch glycolytic fibers were detected in this muscle as expected. No difference either was found regarding all muscle fibers MinFeret diameter distributions, in term of genotype or exercise effect (**Figures F–H**).

## Discussion

The stimulation of NAD^+^ synthesis by the NR vitamin B3, the substrate of NMRK enzymes, has been shown to be beneficial for muscle physiology and mitochondrial function in the context of aging or genetic myopathies in many studies (Mouchiroud et al., 2013; Cerutti et al., 2014; Khan et al., 2014; Frederick et al., 2016; Ryu et al., 2016; Zhang et al., 2016). One exception being a short report that NR mildly decreases exercise performance in rats in a swimming exhaustion test although no muscle phenotyping or molecular analyses were performed to understand this lack of response (Kourtzidis et al., 2016).

We have shown recently that *Nmrk2* levels are strongly induced in the context dilated cardiomyopathy triggered by heart-specific deletion of the SRF transcription factor and that NR-supplemented diet or voluntary wheel running delays the onset of heart failure in this model (Deloux et al., 2017; Diguet et al., 2018). NMRK2, a striated muscle specific kinase has recently been shown to be required in muscle cells, in redundancy with the more ubiquitous NMRK1, to respond to the boosting effect of NR on NAD level (Fletcher et al., 2017). Here, we showed that aged NMRK2 deficient mice present an altered response to exercise training. In the heart, *Cpt1b* and *AcadL* genes, involved in β-oxidation, were reduced with exercise in *Nmrk2*^−/−^ mice. In gastrocnemius muscle, *Nmrk1* levels were reduced in NMRK2 deficient mice, and slow MHC isoform *Myh7* was more strongly induced by exercise in *Nmrk2*^−/−^ mice than in controls. Endurance training reduced the diameter of fibers in control mice but not in *Nmrk2*^−/−^ mice.

In cardiac and skeletal muscle, NAD levels has been described to be highly regulated by NAMPT, that acts as a rate limiting enzyme recycling NAM into NMN to form NAD^+^ (Mori et al., 2014). NMRK1 and NMRK2 enzymes were identified more recently as alternative enzyme using NR as an alternative precursor to salvage NAD^+^ levels (Bieganowski and Brenner, 2004). Here we show that global steady tissue levels of NAD are not altered in the heart and skeletal muscle despite the absence of NMRK2. This result is in agreement with a recently published study in young *Nmrk2*^−/−^ mice that also reported there was no alteration of global NAD levels in quadriceps muscle in the KO mice in comparison to controls, with concentrations around 400 pmol/mg of tissue, which are identical to our findings in 18 month-old mice (Fletcher et al., 2017). In contrast, however, the authors did not observe a difference in term of *Nmrk1* expression in young *Nmrk2*^−/−^ mice, when we found a clear reduction at 18-month of age. However *Nampt* expression is maintained at this age in *Nmrk2*^−/−^ mice, which is apparently sufficient to sustain global levels of NAD. Interestingly, while NAD levels were strongly reduced in the skeletal muscle-specific *Nampt*^−/−^ mice that were intolerant to exercise, NR had a potent rescuing effect on exercise intolerance, fiber size and ATP production despite having no impact on total NAD tissue levels and very minor effect on mitochondrial NAD pool (Frederick et al., 2016). These finding show that the NMN produced from NR by NMRK enzymes is not equivalent to the NMN produced by NAMPT to synthesize NAD. So, while it is clear now that NMRK2 is dispensable for the maintenance of global tissue NAD levels, it suggests that NMRK2 is involved in the synthesis of a subfraction of the NAD pool that is efficiently used by the muscle cells.

Skeletal muscle adaptations to exercise varies depending on the type of exercise. To focus on endurance exercise, different studies have clearly demonstrated in human that endurance training induces a switch from glycolytic to a more oxidative phenotype (Andersen and Henriksson, 1977). In young rats, 10 weeks running program triggers a similar shift toward slower oxidative isoforms of myosin in plantaris muscle, when no shift was reported in soleus muscle (Fitzsimons et al., 1990). Here, we show that in aged mice, treadmill running exercise increases the expression of *Myh7* gene, which encodes a slow contractile isoform of myosin heavy chain (MHCI) that uses less ATP per unit of work because of its lower myosin ATPase activity, relative to fast myosin isoforms, and is thus more metabolically efficient in the context of endurance training. Interestingly, the increase in *Myh7* expression was significantly stronger in *Nmrk2*^−/−^ mice compared to controls, suggesting that NMRK2 signaling is normally blunting the expression of slow myosin in muscle or reciprocally that the lack of NMRK2 reults in a higher need for the muscle to reduce the energy required for contraction. We also found that endurance exercise led to a reduction of the mean diameter of muscle fibers in the plantaris but not the soleus of control mice whereas this phenomenon did not occur in *Nmrk2*^−/−^ mice. It is important to notice that *Nmrk2* expression is 2 times higher in plantaris muscle compared to soleus (Fletcher et al., 2017), which may explain while it function is more easily put in evidence in this muscle.

Considering the emerging role of skeletal muscle in autocrine and paracrine signaling, we also analyzed the expression of several myokines known to be activated by exercise. Among them, IL-15 is a cytokine secreted by skeletal muscle that promotes endurance adaptations via a stimulation of the oxidative energy metabolism and the Sirt1/PGC1a axis in young mice (Quinn et al., 2013). IL-15 levels are increased by exercise in humans (Rinnov et al., 2014). On the other skeletal muscle aging and sarcopenia are associated with low levels of IL-15 (Yalcin et al., 2018). Here, we show that exercise reduced IL-15 mRNA level in aged control and *Nmrk2*^−/−^ mice. On the other hand, we showed that the *IL-15* gene expression is upregulated at baseline in the soleus of *Nmrk2*^−/−^ mice. Since we observed a trend to have thinner muscle fibers in plantaris but not soleus muscle of *Nmrk2*^−/−^ compared to controls, it suggests that the local increase in IL-15 production in the soleus may contribute to preserve muscle mass. The link between NMRK2-dependent NAD metabolism and IL-15 signaling in oxidative metabolism will deserve to be further explored in the future. In the same line, our observation that genes involved in fatty acids β-oxidation (FAO) are abnormally repressed in the heart of *Nmrk2*^−/−^ mice upon training while it is well known that FAO is increased during exercise to respond to the higher energy demand (Jeppesen and Kiens, 2012) suggest a maladaptative metabolic response in absence of NMRK2.

In conclusion, we demonstrated for the first time a phenotype in old *Nmrk2*^−/−^ mice in response to endurance training suggesting that NMRK2 deficient mice are prone to develop muscle dysfunction with aging.

## Author contributions

MM and ZL conceived the study. RD and CT performed the laboratory experiments. AF performed and supervised RD for the endurance training protocol. RD and MM wrote the draft of the manuscript. AF corrected the draft of the manuscript. All authors have read and approved the final manuscript.

### Conflict of interest statement

The authors declare that the research was conducted in the absence of any commercial or financial relationships that could be construed as a potential conflict of interest.
